# Human ex-vivo lung mechanics differ under positive- and negative-pressure ventilation

**DOI:** 10.1186/s12931-026-03518-4

**Published:** 2026-01-26

**Authors:** Kathrine A.M. Quiros, Crystal A. Mariano, Matthew Shankel, Talyah M. Nelson, Mona Eskandari

**Affiliations:** 1 Department of Mechanical Engineering, University of California, Riverside, CA USA; 2 BREATHE Center, School of Medicine, University of California, Riverside, CA USA; 3 Department of Bioengineering, University of California, Riverside, CA USA

**Keywords:** Lung, Pulmonary mechanics, Compliance, Hysteresis, Positive-pressure, Negative-pressure, Recruitment, Resistance, Ventilator-induced lung injury

## Abstract

Clinical and mechanical studies reveal key differences between positive- and negative-pressure ventilation, yet their equivalencyPlease check if the authors and their affiliation are presented and indicated correctly. remains a topic of debate. This debate is perpetuated by contradictory reports from a variety of small- and large-scale animal models. While the usage of animal models has been transformative with incomparable advantages for advancing research efforts, they partially limit the translatability of findings to clinical settings and the accurate assessment of ventilation mode dependencies. For the first time, we inflate ten ex-vivo donated cadaveric human lungs with both positive- and negative-pressure to evaluate mechanistic differences between these ventilation modes and subsequently analyzed the obtained pressure-volume curves using our custom electromechanical ventilator. We report end-inspiratory metrics with disparate end-expiratory and plateau pressures, dynamic compliances, resistance, and viscoelastic metrics between ventilation modes. These rare human lung findings are partly consistent with past animal studies, which reported matched peak pressure-volume behaviors with differing inflation compliances between positive- and negative-pressure ventilation. Differing deflation compliance and end-expiration pressures have not been demonstrated under animal models, highlighting the need for human organ testing. Results suggest distinct recruitment patterns between ventilation modes, providing mechanical insights linked to reports of better oxygenation in negative-pressure ventilation and potential contributors to ventilator-induced lung injury in positive-pressure ventilation.

## Introduction

Artificial ventilation was documented in 1543 by Andreas Vesalius who performed rudimentary positive-pressure ventilation (PPV) seemingly for the first time [1]. Since then, there has been no consensus on whether positive- or negative-pressure ventilation (NPV) is ideal [2–8]. These two ventilation modes differ primarily by how the pressure gradient responsible for lung inflation is initiated; PPV is stimulated via the direct introduction of air into the trachea and airway network, while NPV is accomplished by the application of a negative pressure to the pleural space (i.e., lung periphery). Theoretically, if the lung was perfectly elastic, the manner in which the pressure gradient is achieved should not alter the tissue’s inflation and deflation mechanics, however, numerous studies indicate both clinical and mechanical differences between PPV and NPV including: hemodynamics, oxygenation, cardiac performance, injury, aeration, pulmonary vascular resistance, airway resistance, and elastance [3, 9–14]. However, these differences are not consistently observed amongst animal studies, reinforcing the controversy [5, 6, 9, 15, 16].

The lack of standardization in respiratory studies could be contributing to the differing results as prior mechanical investigations of PPV versus NPV were conducted on a variety of small- and large-scale animal models [4, 5, 14, 17–19].Therefore, the major absence of studies considering disparate ventilation mode mechanics on human lungs is a foundational oversight cause by the extraordinarily restricted availability of human donor lungs. While animal studies indicate differing recruitment mechanisms between PPV and NPV [4, 14, 17, 20, 21], their clinical applicability is limited given the mechanical and physiological differences between human and non-human pulmonary structures [22–28].

Three animal models have primarily been investigated in prior studies: murine, porcine, and ovine lungs. Murine models are an excellent avenue for exploring lung pathologies as many genetically engineered phenotypes exist and induced-disease models are widely adaptable. Yet, the anatomical differences between the murine and human lung – such as lung size, alveolar size, cartilage content, and airway branching patterns – limit translatability [29]. Ovine and porcine lungs are preferred due to anatomical and physiological similarities to human lungs, however, airway branching patterns and lobe segmentation differ [22, 30]. Computational models demonstrate the importance of the mechanical contributions of airway resistance and lobular sliding suggesting the variation between animal and human lungs could affect organ-level mechanics [31–36]. As a critical addition to the growing body of literature that describes the disparate effects of positive- and negative-pressure on lung mechanics, this study seeks to investigate the isolated inflation mechanics of human donor lungs under PPV and NPV for the first time.

Understanding the potential mechanical non-equivalency of PPV and NPV is vital as it may provide insights into ventilator-induced lung injury (VILI). VILI is a concerning phenomenon that can occur due to high end-inspiratory volumes (i.e., volutrauma), low end-expiratory pressures (i.e., atelectrauma), inflammation and high oxygen concentrations (i.e., biotrauma), and high pressures (i.e., barotrauma) [37, 38]. To heighten the concern, current lung-protective ventilation strategies used medically cannot fully eliminate VILI. This problem is persistent as recent studies focusing on ex-vivo lung perfusion (EVLP) find that clinical factors indicating lung damage are increased under PPV compared to NPV, thus revealing ventilation mode correlates to VILI [39, 40]. While there are various reasons for this shortcoming, the main issue is that safe ventilation parameters under PPV are patient specific and not fully understood (e.g., cyclic recruitment/decruitment, mechanical power) [38, 41]. A mechanical investigation of the positive- and negative-pressure ventilation of ex-vivo human donor lungs will provide insights into the basis of observed differences and lung damage.

## Methods

### Donor lungs

Donor lungs in this study were obtained through a California organ procurement and transplant center (Institutional Review Board exemption approval HS 20–180). Lungs were packaged on ice and shipped to the testing location where inflation procedures were conducted within 48 h of organ removal. Subject demographics and relevant medical information can be found in Table 1.

Donor organ transplant eligibility is assessed at two key stages before being routed to either a lung transplant recipient or research facility. Preliminary assessment of transplant eligibility occurs prior to the donor entering the operating room and is based on donor health history, imaging, and current pulmonary function. A second and final assessment is made by the transplant surgeon in the operating room at the time of organ retrieval. Oftentimes, transplant surgeons reevaluate lung quality during the harvest process and reject previously eligible lungs for transplant; lungs are then rerouted for research. As demonstrated by Table 1, of the ten donor lungs tested here, six healthy specimens were initially classified as transplant eligible before ultimately being designated for research. Parametric statistical testing indicated no significant difference in the mechanical metrics of preliminarily eligible and ineligible donor lungs, as such, all donor lungs are analyzed collectively.

**Table 1:** Donor demographics

Subject	Sex	Age (y)	Height (cm)	Weight (kg)	BMI	*P*/F	Lung Health History	Preliminary Transplant Classification (Before Organ Retrieval)	Primary Transplant Classification (During Organ Retrieval)
1	F	46	167	57.6	20	586	Smoker (~ 30 pack years)	Ineligible due to smoking history	
2	F	57	160	46.9	19	190	Smoker (~ 30 pack years)	Ineligible due to smoking history	
3	F	17	152	52	22	172	Asthma	Eligible	Eligible (but timed out)
4	M	24	183	197	> 54	56	Non-smoker	Ineligible due to poor pulmonary function	
5	F	32	167	70.3	25	287	Smoker (~ 7 pack years)	Eligible	Ineligible due to declined pulmonary function
6	M	30	175	80.7	26	297	Non-smoker	Eligible	Ineligible due to declined pulmonary function
7	M	34	178	68.1	22	263	Smoker (~ 4 pack years)	Ineligible due to edema and poor pulmonary function	
8	M	37	170	92	32	213	Smoker (~ 1 pack year)	Eligible	Ineligible due to declined pulmonary function
9	M	60	170	88	30	-	Non-smoker	Eligible	Ineligible due to quality
10	M	60	182	94.8	28	174	Non-smoker	Eligible	Eligible (but timed out)

### Experimental procedure

Lungs were placed inside our custom designed ventilation system – previously detailed [42] – for inflation testing. The volume-controlled ventilation system allows for the direct measurement of lung expansion (i.e., lung volume) by accounting for gas compression in the system and in the lung [42, 43]. To best match mechanistic results from PPV and NPV, the primary focus was matched lung volume between the two ventilation modes at 7 ml/kg [44] (7.1 ± 0.67 ml/kg) (IBW, ideal body weight). Cycling was performed at 15 breaths per minute (BPM) to closely mimic nominal ventilation methods [45]. The testing protocol was as follows [27, 46, 47]: preload inflation pressure to 5 cmH_2_O, cyclic inflation and deflation (total of 3 cycles), preload inflation pressure to 5 cmH_2_O, cyclic inflation and deflation (total of 1 cycle), and a final inflation with a two-minute hold. Positive-pressure testing was conducted first to establish the lung volume to which negative-pressure results would then be matched as done previously; past studies showed testing order did not influence results [4, 17]. The maximum lung expansion volume was used to match ventilation modes and was measurable due to the unique construction of our ventilation apparatus [42]. For all reported mechanics, the fourth cycle is analyzed with matching lung volume, peak transpulmonary pressure (P_max_; pressure at max volume) [48], and resulting end-expiratory pressure (P_exp_; pressure at end of deflation) reported (Fig. 2) alongside a representative PV curve with the baseline adjusted to the origin.

### Lung resistance and elastance

Lung resistance and elastance were calculated via the single compartment lung model [49]. The model – described by Eq. 1 – was fit to the transpulmonary pressure data collected after preconditioning by using the corresponding lung volume data and the flow rates. Flow was calculated by differentiating the volume-time data.

1 $$\:P={E}_{L}V+{R}_{L}\dot{V}+{P}_{o}$$

Here, *P* is the transpulmonary pressure, *E*_*L*_ is the lung elastance, *R*_*L*_ is the lung resistance, *V* is the lung volume, $\:\dot{V}$ is the flow, and *P*_*o*_ is the offset pressure. The fit of this model was evaluated by a least square fit between the calculated pressure values and the measured transpulmonary pressure values.

### Compliance and energetics

To evaluate the elastic behavior of the lung at different points of the PV curve, the bilinear inflation and deflation sections of the curves were each fitted with two linear curves [17, 50, 51], as in previous studies: initial and final inflation compliances (labeled C_start_ and C_inf_, respectively), and initial and final deflation compliances (C_top_ and C_def_, respectively); (Fig. 2). The effect of age on these metrics was also briefly investigated. Hysteresis and energy loss were calculated as the area enclosed by the PV curve and the normalized area, respectively [43, 52]. The pressure drop during the two-minute hold was quantified as the percentage drop in pressure from peak inspiratory pressure to plateau pressure [4, 19].

### Statistical analysis

Data is reported as mean ± standard deviation (SD) with individual data points plotted. Paired t-tests were used to statistically evaluate differences between PPV and NPV for all metrics (GraphPad Prism 10; GraphPad Software, San Diego, CA). The significance threshold was set at *p* < 0.05. Model fit was evaluated and the quality of fit (R^2^) is reported.

## Results

Resultant lung volume was matched between PPV and NPV to ensure comparable lung size and account for gas compression (Fig. 2A&B). Statistical analysis of the lung volumes in Fig. 2 C and maximum pressures in Fig. 2D establishes the notable success of the matching protocol between PPV and NPV given the non-significant difference between modes. End-expiratory pressures reported in Fig. 2E, however, were significantly different. Under PPV, end-expiratory pressure varied widely between donor lungs and was highly negative compared to values reported under NPV. Very few donor lungs produced similar end-expiratory pressures between ventilation modes. 

**Fig. 1:**
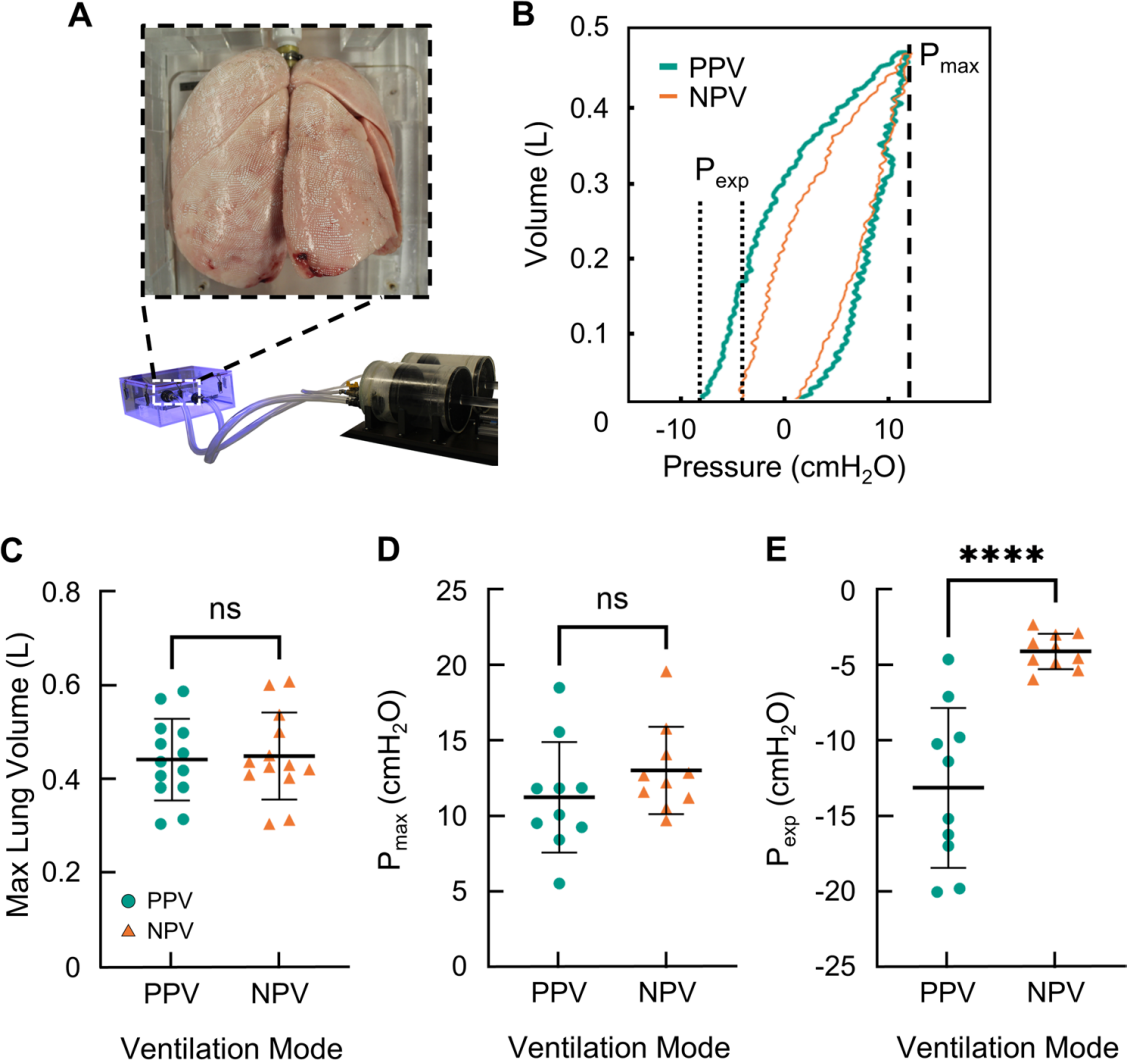
**A** Ex-vivo donor lung inside the custom ventilation system. **B** PV curves (PPV, teal; NPV, orange) demonstrating P_max_ and P_exp_. Notably the **C** matched peak lung volumes and **D** corresponding transmural peak pressure did not statistically differ, demonstrating the success of the matching inflation protocols between PPV and NPV. However, the **E** end-expiratory pressure between PPV and NPV was different

**Fig. 2:**
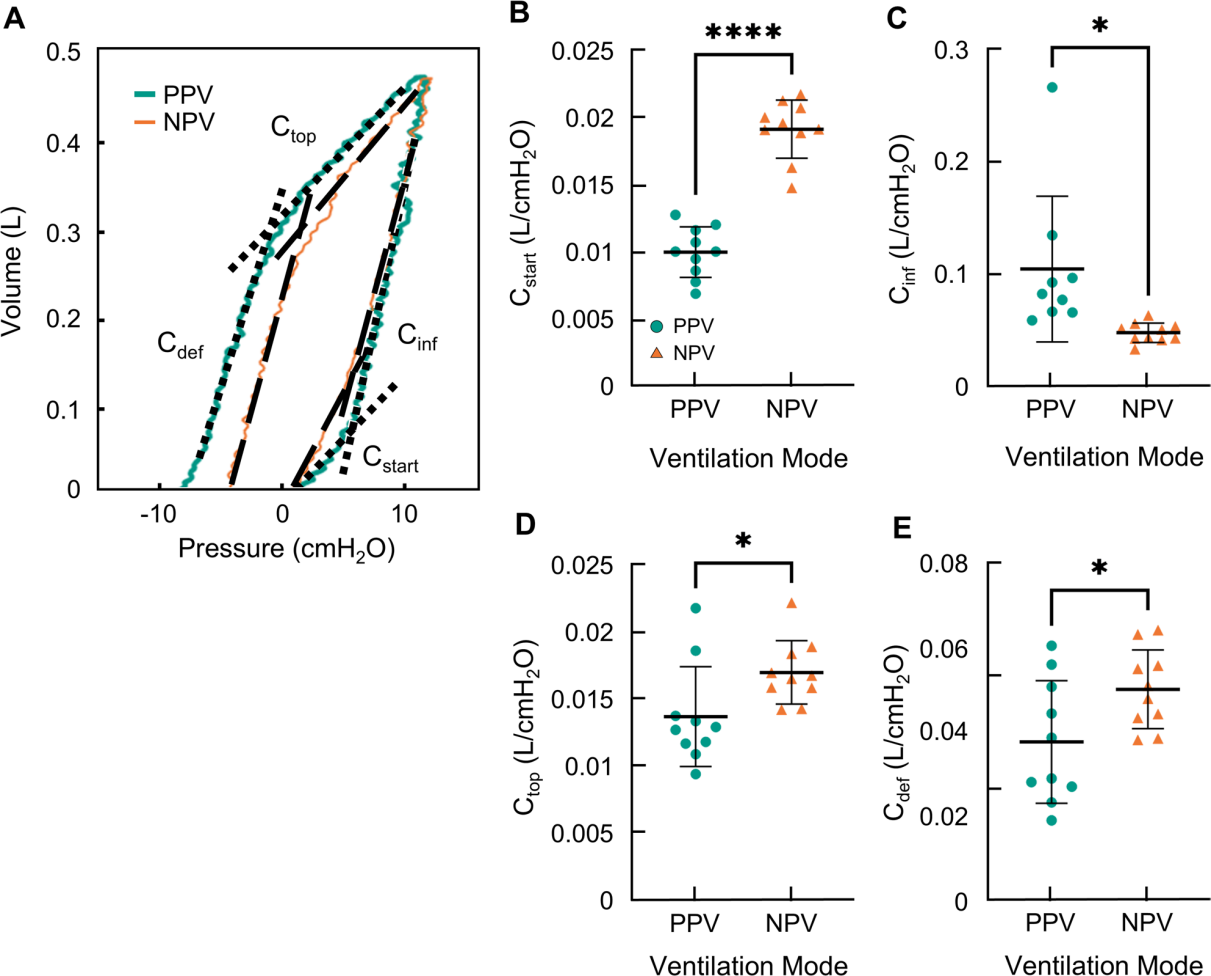
**A** Representative PV curves for PPV (teal) and NPV (orange) illustrating C_start_, C_inf_, C_top_ and C_def_ as black dashed lines. Quantified **B **starting compliance, **C** inflation compliance, **D** top compliance, and **E** deflation compliance with individual lung specimen performances marked

To examine the dynamic behavior of the lung, inflation/deflation slopes of four stages were analyzed (C_start_, C_inf_, C_top_, and C_def_; Fig. 2). The inflation limb of the PV curves was analyzed to assess the dynamic compliances of C_start_ and C_inf_, which were compared between PPV and NPV (Fig. 2A). Initially under PPV, the pressure rises without substantial increases in lung volume resulting in a small C_start_. Contrastingly, under NPV, the volume increases more swiftly resulting in a significantly greater C_start_ (Fig. 2B). After approximately 7 cmH_2_O, the dynamic compliance under both PPV and NPV increases. This end-inflation compliance (C_inf_) (reported in Fig. 2C), is significantly lower under NPV than PPV, with PPV demonstrating incidences of particularly high C_inf_.

The deflation limb was also analyzed for the initial deflation (C_top_) and end deflation (C_def_) (Fig. 2A). The shape of the deflation limbs are similar in both modes but the slopes differ . C_top_ (Fig. 2D) and C_def_ (Fig. 2E) are both found to be significantly higher for NPV compared to PPV.

**Fig. 3:**
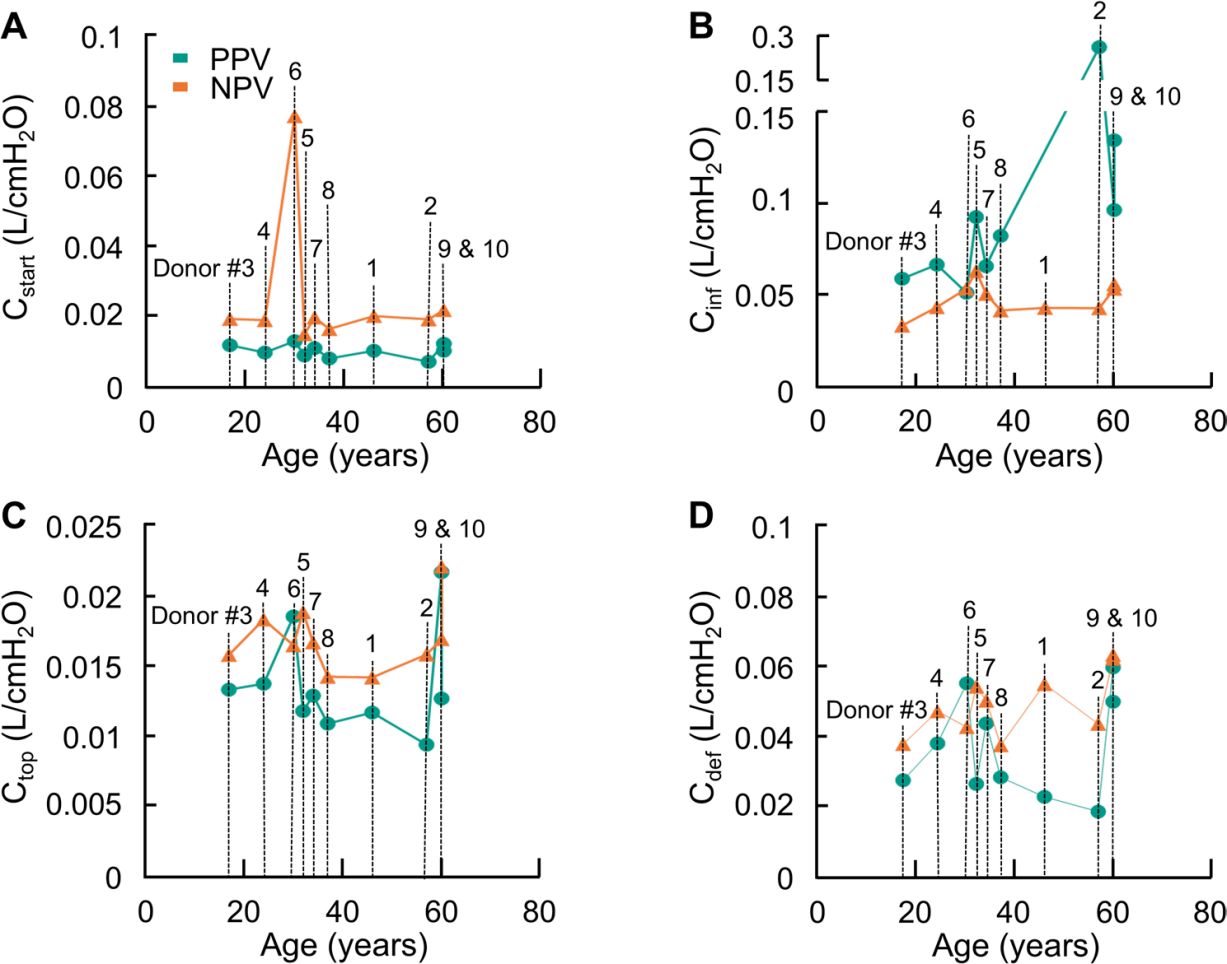
Dynamic compliances are graphed as a function of the age of the donor: **A** C_start_, **B** C_inf_, **C** C_top_, and **D** C_def_. Specific donor number labeling corresponding to Table 1 are indicated in (A)

The effects of age on each dynamic compliance are graphed in Fig. 3. Due to the other confounding factors such as sex, ethnicity, lung history, etc. the authors make no claims as to whether the trends seen here are conclusive. It is worth noting that while C_inf_ appears to increase with age, the two PPV lungs with the highest compliance values also have the most extensive smoking history, preventing accurate assessment.

**Fig. 4:**
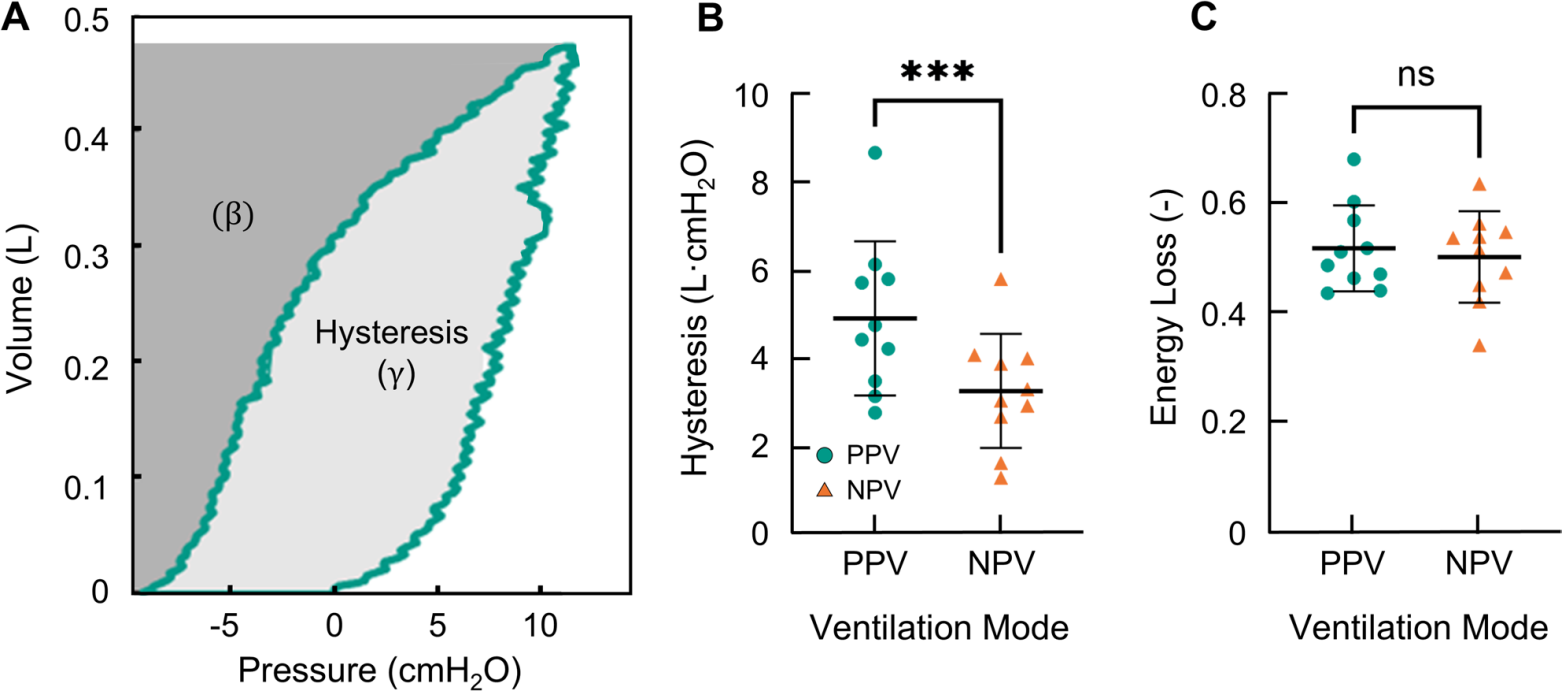
**A** Representative PV demonstrating the calculation of hysteresis ($\:\gamma\:$) and energy loss $\:\left(\frac{\gamma\:}{\gamma\:+\beta\:}\right)$. Each specimens’ **B** hysteresis and **C** energy loss indicated

The energetics of inflation/deflation were investigated as hysteresis and energy loss (Fig. 4A). The hysteresis was reduced under NPV compared to PPV (Fig. 4B). Figure 4C shows that the energy loss was not significantly different between PPV and NPV, despite the difference in hysteresis.

**Fig. 5:**
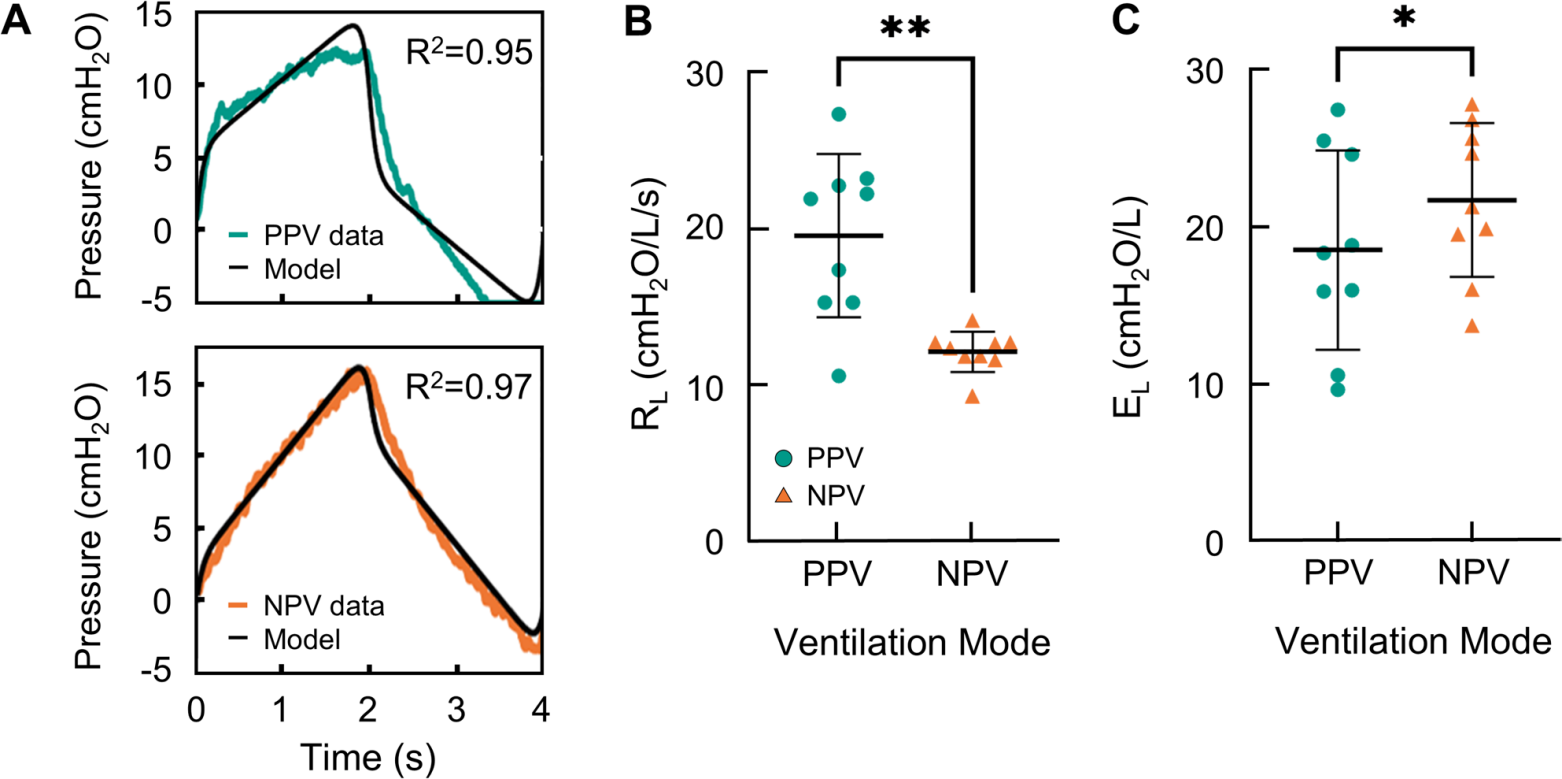
**A** Representative sample measuring temporal pressure evolution after three initial preconditioning cycles with P(0) set to zero (teal, PPV; orange,NPV). The single compartment model was fitted with the respective recorded volume data and the model’s output pressure data is graphed (black). Lung specimens’ **B** resistance and **C** elastance measures for PPV and NPV is shown

To analyze the resistance and elastance of the lungs, the single compartment model was fitted to the lung volume test data. Figure 5A demonstrates the model fit where orange is the NPV pressure data, teal is the PPV pressure data, and black is the pressure resulting from the model. The model fit both data sets similarly, where the averaged fits were R^2^ = 0.94 ± 0.03 and R^2^ = 0.94 ± 0.01 for NPV and PPV, respectively. From the model, R_L_ was calculated, and individual data points are marked on Fig. 5B. Statistical analysis indicated that R_L_ was significantly lower under NPV compared to PPV. Figure 5C reports that E_L_, which was also extracted from the model, was higher under NPV.

**Fig. 6:**
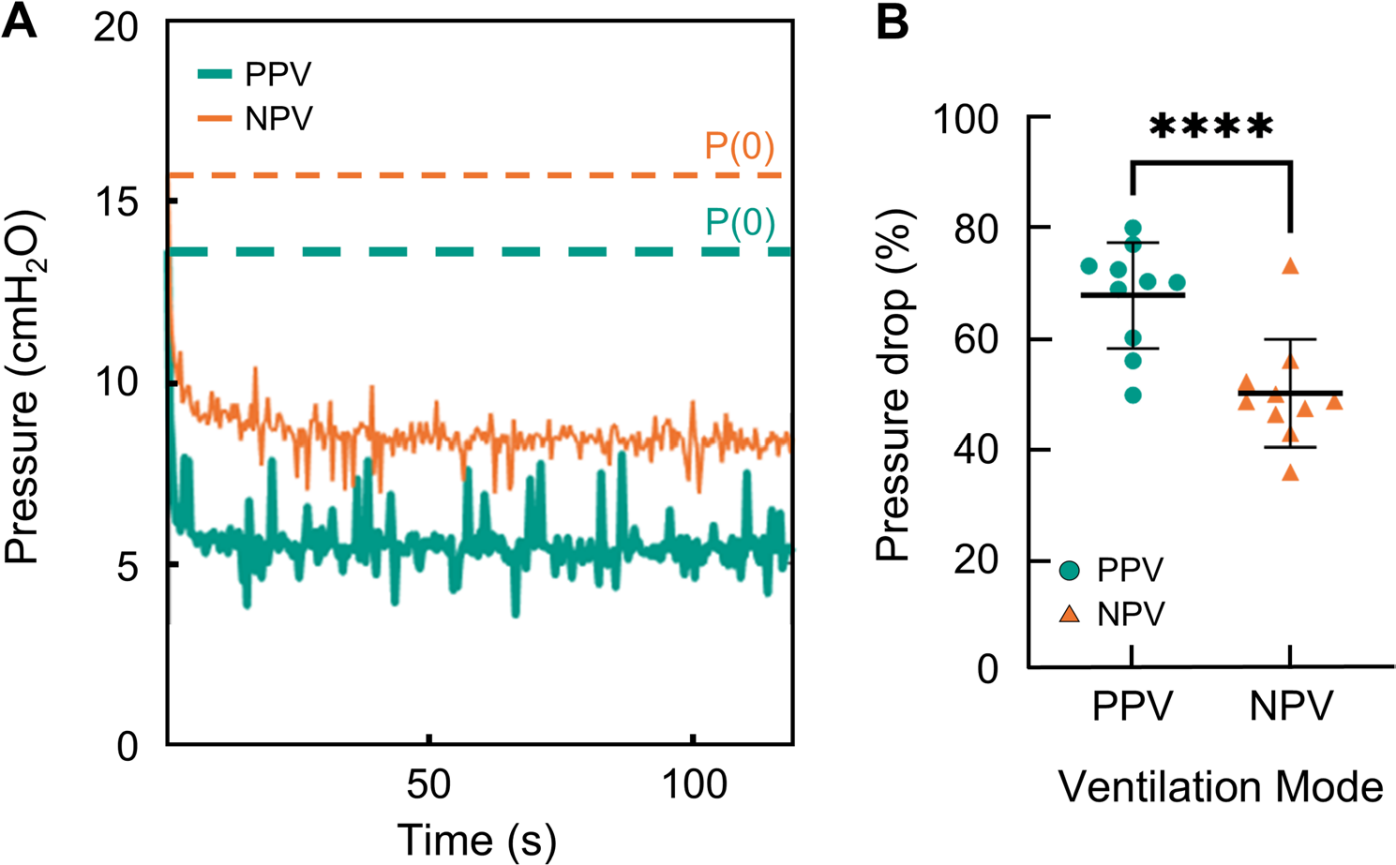
**A** Representative measured pressure data collected during the two-minute hold under PPV (teal) and NPV (orange). Initial pressure values are indicated with horizontal dashed lines in the respective colors to demonstrate pressure drop **B** The pressure drop (percent relaxation) is reported

The pressure relaxation over a two-minute hold at maximum inflation was analyzed. Representative pressure curves for PPV (teal) and NPV (orange) are shown in Fig. 6A with the initial pressure marked by a dashed line. The pressure drop from peak-inspiratory pressure to plateau pressure (Fig. 6B) was increased under PPV compared to NPV.

## Discussion

We investigated mechanistic differences of ex-vivo human donor lungs ventilated using positive- and negative-pressure under normative ventilation conditions (7 ml/kg IBW, 15 BPM) [44, 45] for the first time. Results averaged from a donor pool of ten human lungs (Table 1) showed matched peak inflation mechanics in agreement with past studies [4, 5, 14] (Fig. 1C & D). Testing also revealed differences in inflation and deflation pathways, end-expiratory and plateau pressures, resistance, and energy requirements between PPV and NPV. Inflation and energetic differences are in line with results from large-scale animal models and indicate recruitment mechanisms vary between ventilation modes [4]. While this demonstrates large-animal models more closely reflect human lung mechanics, especially compared to small-scale animal models, it is important to note that the damaging deflation compliance differences noted here are not present in animal studies [4]. Therefore, given the use of rare human donor lungs, these findings are highly relevant overall and particularly for the development and assessment of EVLP devices. Potential physiological explanations and implications of these ventilation mode dependencies are discussed in addition to connections to VILI (i.e., atelectrauma and barotrauma).

### Recruitment

The pressure and volume data collected during inflation are distinctly different between positive- and negative-pressure ventilation. Here, pressure initially builds under PPV without significant volume variation until a critical pressure threshold, ~ 7 cmH2O, where the lungs rapidly expand. This critical pressure occurs at a lower pressure here in human donor lungs than in porcine samples (20 cmH2O) indicating differences in recruitment requirements between human and non-human lungs [4]. This behavior is further quantified by the two dynamic compliances C_start_ and C_inf_, where at the outset of inflation, C_start_ is low in PPV followed by a high C_inf_. Under NPV, the slopes of these regions are significantly disparate compared to PPV. Specifically, C_start_ is greater under NPV as the lung expands more at lower pressures and C_inf_ is lessened under NPV. This significant variation in inflation compliance was previously reported in porcine samples where PPV and NPV were implemented and compared under similar experimental testing conditions at higher volumes [4]; lung elastance and recruitment were suggested to be the primary components contributing to this inflation discrepancy as increased compliance (C_inf_) is indicative of late-stage recruitment [53]. Our results support this claim, and additionally suggest that C_start_ values indicate initial aeration is improved under NPV compared to PPV, in agreement with what has been observed in rabbits [10]; C_start_ is thought to be an indicator of the number of “lung units’’ available (i.e., aeration levels) at the onset of inflation [50, 54, 55].

To further investigate alveolar recruitment, we analyzed lung resistance, which is linked to alveolar recruitment [56]. Here we report that lung resistance was dramatically increased under PPV indicating decreased alveolar recruitment in that mode. This finding supports that recruitment patterns differ between ventilation mode and is thus responsible for inflation discrepancies, supporting previous hypothesizes [54, 56–60].

Why this delayed recruitment pattern does not occur in NPV is unclear, as both positive- and negative-pressure protocols were identical (excluding the ventilation mechanism), with matched peak volumes and pressures (Fig. 1C-D). Given the discrepancy occurs immediately, the reference-state of the organ before inflation should be considered. In both PPV and NPV the respective loading pressure is initially set to 5 cmH_2_O under the corresponding mechanism (i.e., positive- or negative-pressure) and then the test cycle (i.e., fourth cycle) is performed; included figures do not reflect preload as PV curves are translated to origin. The 5 cmH_2_O preload was selected based on the reference literature and is thought to fully open collapsed airways before inflation testing. This ensures that the initial recruitment state under PPV and NPV is consistent. It is possible that the efficacy of this preload differs with ventilation mode mechanism (i.e. PPV versus NPV) contributing to the inflation discrepancies [61–63]; however, this theory would require further testing.

Additionally, the increased hysteresis noted in PPV compared to NPV is also indicative of recruitment discrepancies [64], as more energy is required to open collapsed airways and/or alveolus than would be required to expand open lung units [65, 66].

### Resistance

As demonstrated by Fig. 5, we report that lung resistance is increased in PPV compared to NPV. Tissue and airway resistance are both responsible for overall lung resistance and at our testing frequency, the contribution of tissue resistance is elevated and should be considered [67, 68].

There are many factors that affect tissue resistance which should be contemplated to explain PPV and NPV resistance variations including: the elastic fibers (e.g., concentration, architecture, or orientation [69, 70, 71], the alveolar surface tension, surfactant [20], lung volume, and donor age [72]. Notably, given that the same set of lungs are used under PPV and NPV – intentionally designed to avoid confounding factors or any patient specific inconsistencies (i.e., disease, structure) [73] by comparing the lung to itself – with matched volumes between ventilation modes (Fig. 1C), it is unlikely that any of these factors would be responsible for differing resistance results. While these known contributors of tissue resistance are not responsible for observed trends, Dong et al. [14] and Eskandari and Bates [21] describe that an uncharacterized contributor to tissue resistance results from the viscoelastic nature of the tissue, which may result in the NPV versus PPV differences observed here.

Given that tissue resistance cannot wholly explain the observed discrepancies, attention should be shifted to airway resistance [45, 74]. Airway resistance may differ between NPV and PPV as the total cross-sectional area of the airways has been shown to be increased in NPV compared to PPV [14]. This increased cross-sectional area under NPV should reduce the airway resistance in NPV compared to PPV in agreement with what we observe here [75–77]. As suggested by Dong et al., disparate airway diameters may be tied to the differences in flow patterns, as in NPV flow is initialized by distal volume expansion whereas in PPV, flow is initialized by directly loading the large airways [4]. While the overall transmural pressure gradient is matched between ventilation modes, it is possible that this difference in pressure applications results in diverse internal pressure gradients and correspondingly diverse cross-sectional areas and resistance values [14]. Our increased inflation volumes and variable test subjects (i.e., human donor lungs) may explain why investigations of ovine samples inflated with PPV and NPV at a similar rate (0.25 Hz) failed to detect resistance differences between ventilation mode at these low pressures despite noting differing airway diameters [14]. Differing airway diameters under PPV and NPV in the human lungs should be a topic of future work particularly in an EVLP setup which introduces other factors that may affect distal airway diameter.

### Ventilation-induced lung injury

The negative end-expiratory transpulmonary pressure observed here in both PPV and NPV (Fig. 1E) has significant clinical relevance, given this metrics’ connection to VILI. This detrimental phenomenon is observed during the ventilation of ARDS patients despite positive end-expiratory pressure (PEEP) implementation [78] and indicates damaging alveolar/airway collapse (i.e., atelectrauma) [79]. The noted negative end-expiratory pressure here is destructively lower in PPV compared to NPV, which has not occurred in past animal-studies [4]. As damaging end-expiratory pressures are characteristic of PPV and necessitate intervention (i.e., PEEP), such negative end-expiratory pressures were not surprising. However, given animal studies have not shown differences in end-expiratory pressures between ventilation modes, this ventilation dependence is unexpected. Historically, PEEP was developed during the rising use of positive-pressure ventilation [80] but was never implemented in older negative-pressure ventilation devices [81] tentatively suggesting a connection to this dependency. The absence of these findings in animal studies emphasizes the importance of such human donor lung testing.

Given our matched peak behaviors, the observed differences in end-expiration pressures must be the result of the differing deflation paths (Fig. 2), and may indicate differing aeration levels mid-expiration as seen previously [10]. Furthermore, negative end-expiratory pressures coupled with increased C_inf_ in PPV suggest increased cyclic recruitment and decruitment of alveoli, a known cause of VILI [79]. While more research is needed on human donor lungs to parse out the mechanisms responsible for end-expiration variances, the difference in requirements of positive- and negative-pressure devices (i.e., PEEP) coupled with the differing end-expiratory pressures seen here may provide insights into VILI and indicate the differing deflation patterns may be responsible for atelectrauma. This is a principally relevant finding for EVLP, which results in alveolar damage under cyclic positive-pressure inflation, and it would be interesting to further evaluate the deflation mechanics from EVLP devices [40].

Barotrauma is another form of ventilator-induced lung injury resulting from invasive and non-invasive positive-pressure ventilation [82]. This damage occurs with excessive peak-inspiratory pressures or when plateau pressures exceed 30 cmH_2_O. Employment of protective tidal volumes prevented undue peak pressures within our study [82, 83], yet, plateau pressures were investigated and found to differ between ventilation modes as evidenced by the increased pressure drop in PPV (Fig. 6). Airway resistance, stress relaxation, and pendelluft are all known contributors to this pressure drop; pendelluft typically being the least impactful [84] and resistance characteristically being the most impactful [84, 85]. While separating out contributions of pendelluft, tissue relaxation, and resistance, is challenging [86–88], it is likely that airway resistance is predominately responsible for the increased pressure drop in PPV given our resistance values (Figs. 5 and 6). However, given the disparate heterogeneity of PPV and NPV [4], the effects of internal air redistribution on the pressure decay should not be dismissed. Results of approximate alveolar pressure (i.e., plateau pressure) indicate more air inhabits alveoli in NPV compared to PPV [84, 85, 89], which may be indicative of increased aeration and improved oxygenation [3, 10, 90]. However, as the pressures measured in this study are well within the range of safe ventilatory pressure [82, 83] and evaluation of aeration levels via imaging was not possible, tangible claims cannot be made about the effect of ventilation mode on barotrauma or aeration levels without comparison at higher peak pressures and further analysis.

### Age and smoking history

While the limited availability of human donor lungs restricts our ability to accurately assess the effects of age on lung mechanics, the dynamic compliance data and specifically C_inf_ values highlight the distinct increases in PPV compared to NPV. This behavior is most drastic for lungs sourced from donors with extensive smoking history and the elderly. A larger study investigating this distinct discrepancy between PPV and NPV in smoker lungs and never-smoker lungs, as well as an age-focused study, would be of value to the research community.

### Limitations

Many challenges and restrictions accompany the testing of human donor organs. First and foremost, the availability of donor organs is limited. To ensure that enough lungs were tested to allow statistical comparison, exclusion criteria were minimized, resulting in varied donor backgrounds (Table 1). This impedes the ability to make claims about specific donor characteristics such as age, occupation, lifestyle (e.g., smoking history), and health. Additionally, it is possible that the effect of ventilation modes may vary depending on the pathology and age of the lung. The ten donor lungs included in this study were tested over multiple years. We anticipate a wider pool of donor lungs in the future, wherein we will parse out these dependencies.

The sequential testing of PPV and NPV was not randomized to allow consistent matching protocols (i.e., match NPV lung volume to resulting PPV lung volumes). This decision was based on our previous evaluation and that of other studies which demonstrate the ventilation order of PPV and NPV do not significantly affect resulting mechanics, if preconditioning and resting are implemented [4, 14].

Testing in this study was conducted ex-vivo to isolate lung mechanics. This is highly relevant given the rise of EVLP, which repairs ex-vivo lungs for transplant [39, 40, 44, 91]. However, it does not allow for the evaluation of thoracic cavity mechanics nor oxygenation, which are important for patient applications and should be considered when extrapolating our conclusions to the clinical setting [59, 92].

While we make connections to EVLP studies, we do not perfuse or warm the lung during testing. Testing the organ at room temperature may reduce the diameter of the airways making them less efficient. It is possible that the lack of perfusion may also alter resulting mechanics [93]; however, such an affect is expected to be minimal, given that the analysis of the mechanics of each lung is referenced to itself between PPV and NPV and because both ventilation modes are subjected to the same experimental conditions.

## Data Availability

Due to the inclusion of human cadaveric specimens and the need for institutional approvals (e.g., IRB), the data will be made available upon reasonable request using the contact form at bmech.ucr.edu.
